# Cytokines, Adhesion Molecules, and Matrix Metalloproteases as Predisposing, Diagnostic, and Prognostic Factors in Venous Thrombosis

**DOI:** 10.3389/fmed.2018.00147

**Published:** 2018-05-22

**Authors:** Knut A. Mosevoll, Silje Johansen, Øystein Wendelbo, Ina Nepstad, Øystein Bruserud, Håkon Reikvam

**Affiliations:** ^1^Department of Medicine, Haukeland University Hospital, Bergen, Norway; ^2^Section for Hematology, Institute of Clinical Science, University of Bergen, Bergen, Norway

**Keywords:** venous thrombosis, inflammation, cytokines, adhesion molecules, matrix metalloproteases

## Abstract

The inflammatory response is a well-established part of, and a prerequisite for, venous thrombosis. To better understand the pathophysiology of venous thrombosis and to identify improved diagnostic biomarkers, further studies of the relationship between inflammation and coagulation are needed. We review previous studies concerning inflammatory biomarkers in venous thromboembolism, in particular cytokines, soluble adhesion molecules and matrix metalloproteases as predisposing, diagnostic and prognostic factors in venous thrombosis. Elevated cytokines and genetic alterations coding for cytokines are found in several patient cohorts which indicate that cytokines are involved as predisposing factors in venous thrombosis development. Increased levels of pro-inflammatory cytokines are detected both in animal models and in patients with acute venous thrombosis and clinical trials, although currently without evident diagnostic value. Adhesion molecules are crucial in the development of venous thrombosis, especially P-selectin seems important in initiating leukocyte accumulation and adhesion to endothelium for subsequent platelet accumulation. Several studies have demonstrated increased soluble P-selectin levels in patients with venous thrombosis, emphasizing its potential role as diagnostic marker and also as a therapeutic target. Matrix metalloproteases are essential effectors during venous thrombosis resolution and may impact vessel wall fibrosis, and together with their natural occurring inhibitors are crucial in acute and chronic thrombosis pathophysiology. Furthermore, studies in animal models of venous thrombosis have demonstrated anti-inflammatory treatment to be effective in terms of thrombus resolution and reduction of vessel wall damage, without increase in bleeding risk during the course of treatment. Thus, soluble mediators should be further investigated both as possible biomarkers and therapeutic targets in venous thromboembolic disease.

## Introduction

Venous thromboembolism (VTE) is one of the most common haematological conditions, associated with increased morbidity and mortality, accounting for over 500,000 deaths per year in the European Union (1). Deep vein thrombosis (DVT) and pulmonary embolism (PE) could be difficult to diagnose, with undiagnosed VTE therefore representing an increased risk of death (1, 2). Improved risk stratification and diagnostic tools are important measures for VTE treatment and prevention (3).

Established risk factors for VTE include familial thrombophilia and acquired factors such as malignancies, previous VTE, reduced mobility, trauma or surgery, old age, pregnancy, heart failure, myocardial infarction, ischaemic stroke, obesity and use of oral contraceptives (4–6). Several of these risk factors represent inflammatory conditions (7). Emerging evidence suggests infection as a more important risk factor for VTE than previously recognized, and the coagulation system may play a major role in immune defence (8, 9). To better understand the pathophysiology of VTE and to identify improved diagnostic biomarkers for venous thrombosis, further studies on the relationship between inflammation and coagulation are needed (10). The term inflammation, derived from the Latin word *inflammatio*, is defined as a complex biological response of body tissues to harmful stimuli such as pathogens, damaged cells or irritants (9, 11–13). It is regarded as a protective response involving the innate and adaptive immune system, various blood cells, vessel wall, and a wide range of molecular mediators derived from the various cells involved in the inflammatory process. The main role of inflammation is to eliminate the initial cause of cell injury, to mediate clearance of necrotic cells and damaged tissues from the original insult and inflammatory process, and to initiate tissue repair. The classical local signs of inflammation are redness, heat, swelling, pain, and loss of function. In addition, severe inflammation also elicits systemic effects, probably mediated, at least in part, by circulating soluble mediators originating from the local inflammatory process.

The triad of increased coagluability, blood flow disturbance and vessel wall changes was postulated already by Rudolf Virchow (1821–1902) (14). However, increasing knowledge regarding the biological process bridging inflammation and coagulation have been established since Virchow's initial postulate. In this review we seek to further discuss the complex relationship between inflammation and VTE.

## Pathophysiology of VTE

Endothelial cells are key regulators of the inflammatory response, as they: (i) form a physical barrier for blood cells and regulate the vascular permeability for immune cells, soluble proteins, electrolytes, and water; (ii) regulate the intravascular coagulation; (iii) regulate the vascular tone and blood pressure through initiation of vasoconstriction/vasodilatation; and finally (iv) release hormones and other soluble mediators, such as cytokines, that initiate and regulate the inflammatory process (15).

Endothelial cells activate, control, and direct leukocytes mainly through their cell surface expression of adhesion molecules and the release of chemotactic chemokines after activation. This enables immunocompetent cells to adhere to the endothelial cells and consequently cross the vessel wall by transendothelial migration (TEM), thus resulting in accumulation of immunocompetent cells at the inflammation site (16). Rapid endothelial activation (i.e., within minutes) is induced by stimuli like histamine and platelet-activating factor (PAF), initiating the expression of preformed adhesion molecules. In contrast, pro-inflammatory cytokines, such as interleukin (IL)-1β and Tumour Necrosis Factor (TNF)-α, induce a slower endothelial activation (i.e., within hours), involving transcriptional activation of adhering molecules and chemoattractants (16). The pathogenesis of VTE differs from that of arterial thrombosis, as venous thrombosis is initiated by intravascular events without exposure of the subendothelial structures (17). In a murine model of DVT inflamed endothelium increased the expression of a wide range of adhesion molecules that attach neutrophils and monocytes to the vessel wall, as an initial step in the formation of thrombus mainly consisting of leukocytes and few platelets (18). A complex interplay between monocytes, neutrophils, platelets, and the coagulation cascade leads to the formation of a thrombin-rich thrombus. Activated monocytes express TF that initiates the extrinsic pathway of the coagulation cascade. Thus, findings from the study suggest that TF expression by monocytes appears to be more important than endothelial expression of TF in triggering the coagulation cascade in DVT.

Both the leukocyte-endothelium and leukocyte-platelet interaction is a prerequisite for thrombosis initiation, shown in mouse-model studies of leukocyte integrin Mac-1, platelet C-type lectin-like receptor (CLEC-2), and vWF (19, 20, 21). Leukocyte integrin Mac-1 mediates adhesion of leukocytes-endothelium via the intracellular adhesion molecule-1 (ICAM-1), and adhesion of platelets via GPIbα, and targeting this adhesion-molecule could represent a possible therapeutic target (21). CLEC-2 is a receptor for podoplanin released from the endothelium during venous stenosis, and subsequently trigger thrombosis formation (20). CLEC-2 deficient mice are protected against thrombosis, and treatment of anti-podoplanin neutralizing antibodies reduces thrombosis size in animal models.

Moreover, neutropenia, genetic knockout of factor XII, and neutrophil extracellular traps (NETs) disintegration protected against the formation of DVT (22). This indicating neutrophil activation as a prerequisite for DVT formation, since neutrophils bind coagulation factor XII and release NETs, which together with platelets activate the coagulation cascade both through the intrinsic and extrinsic pathways (18). The host might prevent vascular occlusion through DNases that helps dissolve the NETs during an inflammatory response, thus control and prevent thrombus formation (23).

## Soluble mediators during inflammation

**Figure 1:**
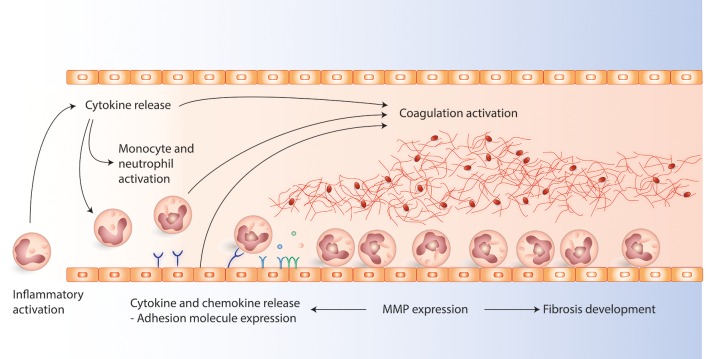
The figure present the main interactions between cytokines, chemokines, adhesion molecules, matric metalloproteases (MMPs), and coagulation activation involved in the pathophysiology during venous thrombosis development. Cytokines are early initiators of inflammation, ameliorating the interactions between leukocytes and endothelial cells. Activated leukocytes and endothelial cells express adhesion molecules which promotes leukocyte attachment to the endothelium which is important in the initiation and development of venous thrombosis. MMPs are involved in fibrosis of the vein walls modulation. In addition MMPs are important modulators of cytokines and adhesion molecules during inflammation as they can alter cytokines and contribute to adhesion molecule shedding.

Cytokines, adhesion molecules, and matrix metalloproteases (MMPs) are some of the key components involved in inflammation, see Figure 1 (12, 16, 24). Cytokines are important for cell-cell communication during inflammation, and they are highly heterogeneous and can be classified based on their function, structure, or based on their mechanism of action in the immune system, i.e., pro-inflammatory, anti-inflammatory or adaptive (12). The main pro-inflammatory cytokines belong to the IL-1, IL-6, IL-17, interferon, and TNF families. The IL-1 family is essential for initiation of the inflammatory cascade (25, 26), and cytokines in the IL-6 family have both immunoregulatory, as well as other systemic effects (27). The main anti-inflammatory cytokines include those in the IL-10 (28) and IL-12 families (29–32).

Adhesion molecules are important mediators of cellular adhesion between leukocytes and endothelial cells and can exist in the membrane-bound as well as the biologically active soluble forms (16). The adhesion molecule family includes selectins (L-, E-, and P-selectins) and various immunoglobulin (Ig) family members [e.g., Vascular Cell Adhesion Molecule 1 (VCAM-1), ICAM-1/2]. E-selectin is expressed by endothelial cells, L-selectin by leukocytes, and P-selectin by endothelial cells and platelets, and they are all induced by pro-inflammatory mediators. Selectins are important for leukocyte rolling, and P-selectin is also involved in thrombus formation and intravascular coagulation during infection (9, 16). Ig family members are of particular importance in firm adhesion and trans-endothelial migration of leukocytes, and both ICAM-1 and VCAM-1 are expressed by endothelial cells (33).

Finally, MMPs have emerged not only as molecules involved in modelling extracellular tissue, but also as important regulators of inflammatory responses, e.g., through their activation and modulation of pro-inflammatory cytokines (24). Matrix metalloproteases (MMPs) and protease inhibitors both interact with cytokines and adhesion molecules at various levels. Hence, one may argue that these mediators should be regarded as functional parts of the cytokine network. MMPs are zinc-dependent enzymes belonging to the metzincin superfamily of zinc endopeptidases that, to date, comprise 24 mammalian proteases (24, 34, 35).

## Cytokines and VTE

A range of risk factors for VTE include inflammatory conditions, gives rise to the hypothesis that elevated inflammatory mediators are risk factors for DVT and post thrombotic syndrome (PTS). Levels of several cytokines are found elevated as part of VTE pathobiology (Table 1), and findings from selected human and animal studies showing the importance of adhesion molecules in VTE are presented in Table 1.

**Table 1:** Cytokines as predisposing factors, diagnostic markers, and prognostic markers in venous thrombosis.

	**Predisposing factor**	**Acute reaction and diagnostic use**	**Effect on thrombus resolution**
IL-1α	−899C/T ↓ SNP: 108 DVT vs. 325 controls (36)		
IL-1β	rs1143634 ↓ SNP in DVT in larger cohort (4) ↔ 506 DVT vs. 1464 controls (37) IL1RN-H5H5 ↑ Leiden thrombophilia study (38)		
IL-4	−589 T allele ↑ SNP: 108 DVT vs. 325 controls (36)		
IL-6	↔ 506 DVT vs. 1464 controls (37) −174 CC ↑ SNP: 108 VTE vs. 325 controls (36) −174 G > C ↑ SNP: 130 DVT^+^ and 190 DVT^−^ (cancer patients) vs. 215 controls (39) −174 GC ↑ SNP: 119 VTE vs. 126 controls (40) −174 G > C ↔ SNP: 128 DVT, 105 PE vs. 122 controls ↔ IL6: 128 DVT, 105 PE vs. 122 controls (41) CC −572 G/C ↑ 140/246 VTE vs. 160/292 controls, respectively (42, 43) ↑IL6, 200 ovarian cancer, predictor for VTE (44) ↑IL6 in 34 VTE 322 patients with diffuse large B-cell lymphoma (45)	−174 G > C ↔ 128 DVT, 105 PE vs. 122 controls (41) ↑ 84 VTE vs. 100 controls (46) ↑ 49 VTE vs 48 controls (47) ↑ 40 DVT^+^ vs. 33 DVT^−^ (7) ↑ 201 DVT vs. 60 controls (48) ↑ abdominal cancer, post-operative [40 DVT vs. 40 non-DVT vs. 40 controls (49) ↔ 181 cases vs. 313 controls (50) ↑ 68 cases vs. 67 controls (51)	↑ 182 recurrent VTE vs. 350 controls (52) ↑ in post-thrombotic syndrome, 49 DVT (53) ↑ in post-thrombotic syndrome, 136 DVT (mice) (54) ↑ in post-thrombotic syndrome, 387 DVT (55) ↑ risk for post-thrombotic syndrome, 110 DVT patients (56) ↑ 201 DVT vs. 60 controls (48) ↔ 181 cases vs. 313 controls (50) ↑43 DVT vs. 43 controls (57) ↑ increased risk for post-thrombotic syndrome, 803 participants SOX trial (58)
CXCL8/IL-8	↔ 506 VTE vs. 1464 controls (37) −251AT ↑ SNP: 119 VTE vs. 126 controls (40) ↑ 474 DVT vs. 474 controls (59)	↑ 49 VTE vs. 48 controls (47) ↑ 40 DVT^+^ vs. 33 DVT^−^ (7) ↔ 181 cases vs. 313 controls (50)	↑ 182 recurrent VTE vs. 350 controls (52, 59) ↔ 181 cases vs. 313 controls (50) ↑43 DVT vs. 43 controls (57) correlation between baseline lumen diameter of the femoral thrombi and IL-8 cytokine (60) ↔ risk for post-thrombotic syndrome, 387 DVT (55)
IL-10	↓ in VTE group in trauma cohort (61) ↔ 506 VTE vs. 1464 controls (37) rs1800872 ↑ SNP IL-10 in DVT cohort (22 413 women) (4) −1082GG genotype ↓ in 660 DVT vs. 660 controls (62) ↑IL10 in 34 VTE 322 patients with diffuse large B-cell lymphoma (45)	↓ abdominal cancer, post-operative (40 DVT vs. 40 non-DVT vs. 40 controls (49) ↔ 181 cases vs. 313 controls (50)	↔ 181 cases vs. 313 controls (50) ↓ 43 DVT vs. 43 controls (57) ↑ increased risk for post-thrombotic syndrome, 803 participants SOX trial (58) ↔ risk for post-thrombotic syndrome, 387 DVT (55)
IL-12p70	↔ 506 VTE vs. 1464 controls (37)		
IL-13	↑ TT genotype: 108 VTE vs. 325 controls (female) (36)		
CCL2/MCP-1	−2518AG ↑ SNP: 119 VTE vs. 126 controls (40)	↔ 181 cases vs. 313 controls (50)	↑ 182 recurrent VTE vs. 350 controls (52) ↑ in post-thrombotic syndrome, 136 DVT (mice) (54) ↔ 181 cases vs. 313 controls (50) ↔ risk for post-thrombotic syndrome, 387 DVT (55).
TNF-α	↑ TNF-α in VTE in cancer cohort (63) ↑ TNF-α and TNFA haplotype in 15 VTE in cancer cohort 157 GI cancer and controls 157 (64) ↑−308A allele 68 patients vs. 62 controls (65)	↔ 49 VTE vs. 48 controls (47) ↑ 201 DVT vs. 60 controls (48) ↑ 68 patients vs. 67 controls (51)	↑43 DVT vs. 43 controls (57)
IFN-γ			↑ IFN-γ enhances thrombus resolution in mice through enhanced MMP9 and VEGF expression in mice (66)
TNFSF4	SNP ↑ (921C > T), ↓ (rs3850641) 344 DVT vs. 2269 controls (67)		
NF-κB		↑ abdominal cancer, post-operative (40 DVT vs. 40 non-DVT vs. 40 controls (49)	
TGF-β1 TGF-β2		↔ 181 cases vs. 313 controls (50)	↔ 181 cases vs. 313 controls (50) ↓ MATS 42 recurrent DVT vs. 84 controls (68)
PDGF		↔ 181 cases vs. 313 controls (50)	↔ 181 cases vs. 313 controls (50)
Multiplex analysis		IL1RA, EGF, HGF CXCL5, CXCL10, and Leptin ↑21 DVT vs. 20 controls (69) IL1-α, IL1-β, IL-2, IL-4, IL-5, IL-6, IL-7, IL-8/CXCL-8, IL-10, IL-12, IL-13, IL-17, IL-22, IL1RA, CCL-3/4/5/11, CXCL-5/10/11, bFGF, G-CSF, GM-CSF, VEGF, TPO, EGF, HGF, and Leptin, IFN-γ CD40L, TNF-α ↔21 DVT vs. 20 controls (69).	

*This table summarizes selected key human and animal studies of c response in venous thrombosis. Arrows indicate the following: the cytokine/genetic polymorphism coding for the cytokine is elevated/more frequent (↑), decreased/less frequent (↓), or unchanged (↔) in DVT cohorts as a predisposing factor (left column), as part of the acute reaction (middle column), or as a risk factor for post-thrombotic syndrome or recurrent DVT (right column). PTS, post thrombotic syndrome; SNP, single nucleotide polymorphism; TIMP, tissue inhibitor of metalloproteases. Control, healthy control*.

### Cytokines in inflammation and predisposition to VTE

Single nucleotide polymorphisms (SNPs) affecting cytokine genes are associated with an increased risk, and others with a reduced risk, of VTE (Table 1) (4, 67, 36, 40, 39). IL-1 cytokines are early inflammatory mediators. SNPs in IL-1α and IL-1β have shown to be associated with reduced risk of DVT, while another SNP for IL-1β is associated with increased risk of DVT (38). IL-6 is an inflammatory mediator later in the cascade, and is maybe the most studied cytokine during inflammation. Also certain SNPs of IL-6 has been associated as a risk factor for VTE (36).

A number of small case-control studies demonstrated increased levels of pro-inflammatory, or decreased levels of anti-inflammatory, cytokines in patients at risk for DVT (61, 63, 45). Although in a larger, prospective population-based case-control study, no such associations were found (37).

Increased inflammation is also a common feature of most of the epidemiologically proven risk factors (70). As previously genetic factors affecting the coagulation system (e.g., factor V, Leiden, and prothrombin mutations) are predisposing risk factors for VTE (4, 41, 71, 72). However, family history in itself is an independent risk factor that could be more useful than genetic testing when assessing the risk for thrombosis (73). This indicates that genetic predispositions are multifactorial and a complex combinations of several genetic traits, in which inflammation could represent a part of the explanation.

### Cytokines as part of the acute reaction and their possible diagnostic use

Increased levels of pro-inflammatory cytokines were detected in animal models of acute venous thrombosis (74). Several inflammatory cytokines like IL-6, IL-8/CXCL-8, and TNF-α are increased during acute thrombosis, although with no evident diagnostic value (7, 40, 59, 47, 46, 49). The focus of most of the studies has been the main cytokines IL-1, IL-6, IL-8/CXCL-8, and IL-10. Increased inflammatory mediators during the clinical course of thrombosis are in line with clinical signs as redness and swelling. However, even broader inflammatory profiles have limited diagnostic value as most differential diagnoses also show extensive inflammatory activity (69).

### Cytokines and thrombus resolution

Inflammation is considered as an essential response during venous thrombosis formation and resolution, and increased levels of inflammatory cytokines seem to have prognostic impact (56, 55, 53). IL-6 could represent a therapeutic target in the prevention of post-thrombotic syndrome, as suggested by a previous study using an animal model of DVT (54).

In conclusion, cytokines and play a role as predisposing factors for VTE, however they are not established as *important* risk factors. Elevated cytokines are found in several studies of VTE patients, although it is difficult to draw clear conclusion of causality of the cytokines role in venous thrombosis in these patient cohorts.

## Adhesion molecules and VTE

Adhesion molecules are part of the formation of the inflammatory response (Figure 1). The P-selectin stored in platelets and endothelial cells, has been proposed as a key molecule in thrombosis and haemostasis (75, 76). P-selectin has been found upregulated as early as 6 h after thrombus induction (77). The binding of P-selectin to its receptor P-selectin ligand-1(PSGL-1) present on the surface of leukocytes and platelets, initiates pro-coagulatory mechanisms (77, 78). Findings from selected human and animal studies showing the importance of adhesion molecules in DVT are presented in Table 2.

**Table 2:** Adhesion molecules as predisposing factors, diagnostic markers and prognostic markers in venous thrombosis.

	**Predisposing factor**	**Acute reaction and diagnostic use**	**Effect on thrombus resolution**
P-selectin	↔ in DVT group in trauma cohort (61) ↔ selectin haplotypes in Leiden Thrombophilia Study (79)	↑, meta-analysis 586 DVT, 1,843 controls (75) ↑, lower extremity: 112 DVT vs. 122 non-DVT ↔, upper extremity: 32 DVT vs. 13 non-DVT (76) ↑ 62 DVT vs. 116 non-DVT (78) ↑ in DVT patients vs. controls (80) ↑ 49 VTE vs. 48 controls (47) ↑ 22 DVT vs. 21 non-DVT vs. 30 controls (81) ↔ 37 DVT vs. 32 non-DVT (82) ↑ 52 DVT vs. 83 non-DVT (83) ↑ platelet expressing P-selectin in post-operative DVT (84) ↑ 89 DVT vs. 126 controls (85) ↑21 DVT vs. 68 non-DVT (69)	↑ in acute DVT predicts post-thrombotic syndrome, 49 DVT (53) ↑ After anticoagulation therapy, possible therapeutic target? (86) ↓ 1 month after DVT: patients with chronic thrombosis vs. in patients with resolved (80) P-selectin inhibition decreases post-thrombotic vein wall fibrosis in a rat model (87) P-selectin inhibition enhances thrombus resolution and decreases vein wall fibrosis in a rat model (88) P-selectin/PSGL inhibitors equal enoxaparin in VTE treatment (77)
ICAM-1		↔ ICAM-1 37 DVT vs. 32 non-DVT (82) ↑ 181 cases vs. 313 controls (50) ↔21 DVT vs. 20 controls (69)	↑ risk for post-thrombotic syndrome, 387 DVT (55) ↑ increased risk for post-thrombotic syndrome, 803 participants SOX trial (58)
VCAM-1		↔ 49 VTE vs. 48 healthy controls (47) ↔ 37 DVT vs. 32 non-DVT (82) ↑ 52 DVT vs. 83 non-DVT (83) ↑ 181 cases vs. 313 controls (50) ↑21 DVT vs. 68 non-DVT 20 controls (69)	↔ risk for post-thrombotic syndrome, 387 DVT (55) ↑ 181 cases vs. 313 controls (50)
E-selectin	↔ selectin haplotypes in Leiden Thrombophilia Study (79)	↔ 37 VTE vs. 32 non-VTE (82) ↑ abdominal cancer, post-operative [40 DVT vs. 40 non-DVT vs. 40 controls] (49) ↔ 28 VTE vs. 92 non-VTE (89)	

*This table summarizes selected key human and animal studies of adhesion molecule response in venous thrombosis. Arrows indicate the following: the adhesion molecule/genetic polymorphism coding for the cytokine is elevated/more frequent (↑), decreased/less frequent (↓), or unchanged (↔) in DVT cohorts as a predisposing factor (left column), as part of the acute reaction (middle column), or as a risk factor for post-thrombotic syndrome or recurrent DVT (right column). PTS, post thrombotic syndrome; SNP, single nucleotide polymorphism; TIMP, tissue inhibitor of metalloproteases. Control, healthy control*.

### Predisposing factors for VTE

The gene for coagulation factor V is genetically closely linked to the genes encoding the selectins. In a study by Uitte de Willge et al. examination of the haplotypes of E-selectin and P-selectin demonstrated no association with DVT risk (79). Proctor et al. observed that serum P-selectin (sP-selectin) and IL-10 had a consistent association, during the first week after an injury, with the presence or absence of VTE. The sP-selectin level difference was not statistically significant between the groups. Those patients who developed VTE had a higher sP/IL-10 ratio, where lower levels of IL-10 accounted for the ratio-difference, statistically significant to those without VTE (61).

### The acute phase reaction and diagnostic use

Currently the biomarker used in VTE diagnostic is D-dimer, which is a non-specific biomarker of VTE, being a fibrin degradation product (76). sP-selectin has been a candidate as replacement of D-dimer as a diagnostic marker. An early study published in 2000 demonstrated increased sP-selectin levels in thrombosis patients (85). The two most promising studies demonstrated high positive predicative value (PPV) of 91/100%, and a specificity of 97.5/96% for establishing the diagnosis of DVT, with Wells score ≥ and sP-selectin ≥90 ng/ml (76, 78). The first study suggested that P-selectin may be both more specific and sensitive than D-dimer as a diagnostic marker at least in specific subsets of patients (78). The subsequent study also demonstrated higher specificity for sP-selectin, however D-dimer in combination with Well's score performed as well as sP-selectin combined with Well's score in ruling out DVT (76).

Changes in platelet expression of P-selectin may also be used as a diagnostic marker of susceptibility to DVT in high risk surgical procedures, demonstrated in a study of female patients undergoing a total knee arthroplasty (84). A small prospective study published in 2003 examined ICAM-1, VCAM-1, E-selectin, and P-selectin in the diagnostic of DVT, and no significant difference were found between the concentrations in patients with DVT vs. controls (82). Increased levels of E-selectin have also been found as an independent risk factor associated DVT in postoperative patients with abdominal malignancy (49), and VCAM levels seem to be higher in DVT patients (83).

### Adhesion molecules and thrombus resolution

Post-deep vein thrombosis venous insufficiency is characterized by vein wall fibrosis, and is central in the development of PTS, where the long term outcome is influenced by early resolution of the thrombus (80). The inflammatory response has been found to affect both thrombus resolution and PTS development (55, 77, 87). P-selectin and D-dimer have been found elevated in the acute phase of DVT, and decrease significantly during the first month (86). The recanalization is inversely related to coagulation markers and fibrinolytic inhibition (53). Increased levels of P-selectin may represent a hypercoagulable state, although it remains unclear if it precedes and promotes thrombosis or is a consequence of thrombosis (86). In patients with a more serve inflammatory response, the endogenic fibrinolysis is inhibited and coagulation stimulated and therefore thrombus resolution is delayed. This is demonstrated by an increased level of IL-6 and P-selectin in the chronic phase, indicating a worse prognosis (53). However, sP-selectin levels are higher 1 month after a thrombosis in patients who resolved their DVT, implying a more active thrombus metabolism in these patients compared to those with chronic thrombosis (80).

A cross-sectional study in a chronic phase after pregnancy-related DVT demonstrated higher levels of ICAM-1 and VCAM-1 in cases compared to controls. However, after univariate analyses these markers where not significantly associated with PTS (50). Though, patients who develop PTS appear to have higher ICAM-1 level then those patients who do not develop PTS (55).

P-selectin is strongly influenced by treatment of oral anticoagulation, illustrated by increased levels within few months after discontinuation of vitamin-K antagonists. Two rodent model studies showed that P-selectin inhibition decreases post DVT vein wall fibrosis (87, 88). It is assumed that the effect is associated with decreased profibrotic mediators, independent of changes in thrombus masses (87). A meta-analysis of non-human primate models confirmed a significant difference in the effect of vein-reopening and inflammation when comparing P-selectin antagonism and saline (78). Observations that thrombus formation and extension may be inhibited, and enhanced resolution when a DVT is treated with P-selectin antagonists highlight the importance of sP-selectin in thrombosis (75, 88).

In conclusion, adhesion molecules are vital for thrombus formation. P-selectin is a possible diagnostic marker for DVT, and might also represent a therapeutic target.

## Matrix metalloproteases and VTE

MMPs are important in inflammatory responses through their regulation of inflammatory mediators, as well as in maintenance of the function and integrity of physical barriers (Figure 1) (24). Animal models have suggested that MMPs are important effectors during VTE resolution and reduce vessel wall fibrosis (66, 90), thus indicating that MMPs represent potential therapeutic targets (54). Findings from selected human and animal studies showing the importance of MMPs in DVT are presented in Table 3.

**Table 3:** Matrix metalloproteases as predisposing factors, diagnostic markers and prognostic markers in venous thrombosis.

	**Predisposing factor**	**Acute reaction and diagnostic use**	**Effect on thrombus resolution**
MMP-9	1,562 C > T ↑ SNP: 130 DVT^+^ and 190 DVT^−^ (cancer patients) vs. 215 controls (39)	↑ in VTE (80)	↑ IFN-γ enhances thrombus resolution in mice through enhanced MMP-9 and VEGF expression in mice (66) Review: the role of MMPs in DVT [mouse models] (90)
MMP-1, 2, 3, 7, 8, 9 TIMP-1/2		↑ MMPs: 201 DVT vs. 60 controls (48)	↑ MMP-1/8: 47 of 201 DVT developing PTS (48)
MMP- 2, 3, 7, 8, 9		↑21 DVT vs. 20 controls, ↔21 DVT vs. 68 non-DVT (69)	

This table summarizes selected key human and animal studies of MMP response in venous thrombosis. Arrows indicate the following: the MMP/genetic polymorphism coding for the cytokine is elevated/more frequent (↑), decreased/less frequent (↓), or unchanged (↔) in DVT cohorts as a predisposing factor (left column), as part of the acute reaction (middle column), or as a risk factor for post-thrombotic syndrome or recurrent DVT (right column). PTS, post thrombotic syndrome; SNP, single nucleotide polymorphism; TIMP, tissue inhibitor of metalloproteases. Control, healthy control

### The role of MMPs as predisposing factors for VTE

SNPs affecting MMP-9 and IL-6 have been evaluated as predisposing factors for DVT in cancer patients (39). When comparing healthy controls with cancer patients with DVT, both SNPs were associated to DVT. Distribution of the SNPs was similar between healthy controls and DVT negative cancer cases. The results indicate that the GG and CC genotypes, respectively for both SNPs, are associated with increased risk of DVT in cancer patients by inducing the release of IL-6 with subsequent increment of MMP-9.

### MMPs in acute VTE and post thrombotic syndrome

Studying PTS, one study aimed to quantify the change in vein wall thickness in patients who failed to resolve DVT within 6 months, and whether differences in levels of inflammatory proteins associated with venous remodelling (80). Results demonstrated increased levels of MMP-9 antigen in thrombosed patients compared with controls. Other biomarkers such as D-dimer and P-selectin were higher in thrombosed patients at diagnosis, although not significantly different at 6 months. P-selectin gene expression were higher in leukocytes from patients with chronic DVT compared with those who resolved within 1 month after diagnosis.

In another study the expression of inflammatory biomarkers in the early phase of DVT and their correlation with the onset of PTS was evaluated (48). Samples were harvested from 201 patients after their first episode of DVT over a period for 18 months, and from 60 patients without DVT. Analyses of inflammatory biomarkers demonstrated high plasma levels of MMPs and cytokines during the acute phase after DVT. Patients with PTS demonstrated higher levels of MMP-1 and MMP-8 than patients without PTS, implying a close relationship between DVT, the individual risk of PTS and specific biomarkers such as MMPs.

In conclusion, few studies exploring the role of MMPs in VTE are performed. MMPs are elevated during VTE and PTS, although also during other inflammatory conditions, and so far not proven value as diagnostic markers (69).

## Conclusions and future perspective

Inflammatory mediators are part of several cascades in the pathophysiology of VTE; predisposing factors, initiation and prolongation of acute thrombosis and resolution. Especially cytokines have been implicated as a *predisposing factor* in several studies, while adhesion molecules and MMPs are to a lesser degree studied (Tables 1–3). High quality studies demonstrating the effect of inflammatory mediators as predisposing factors of thrombosis are difficult to design. However, the performed studies combined with the knowledge that most risk factors of VTE involves inflammation, indicate an important role of inflammatory mediators as risk factors for thrombosis. The complexity of the genetic predisposition of most human disease hampers to decipher the exact impact of different genetic and environmental factors (91). The focus on family history instead of genetic analysis in clinical assessment is a reasonable approach given this genetic complexity (73, 92). Until further studies have given utterly information, genetic tests involving inflammatory mediators should not be part of routine clinical practice (92).

Several case-control studies and selected animal studies clearly demonstrate the role of the inflammatory response in VTE (Tables 1–3). Despite this, few inflammatory mediators have shown utility in *diagnostics* compared with traditional biomarkers; D-dimer and CRP being the most important ones. The only inflammatory marker shown almost as helpful as D-dimer in diagnostic work up is sP-selectin, although has so far not proven to add definitive diagnostic value. As prognostic factors, the studies presented in Tables 1–3 are indicating higher inflammatory mediator levels can be used to identify patients of risk of PTS. Larger thrombotic burden is a reasonable explanation of these finding.

The intertwined effect of inflammation and coagulation is an important aspect of treatment of VTE. Inflammation as a target is responsible for parts of the effects of low molecular heparin as it is reducing TNF-α expression (93). Furthermore, statins have anti-inflammatory effects believed to be favourable for thrombosis resolution (94–97). Other modulations of the inflammatory response are explored in animal models as possible treatments. Increased levels of cytokines such as IL-8, CCL2 are favourable for thrombus resolution (98, 99), and impaired inflammatory response by cytokine receptor deletion or neutropenia also reduces thrombus resolution (100, 101). This leads to the assumption that the inflammatory response is a prerequisite not only for the thrombus formation, but also the resolution, although the results from using cytokine stimulation are conflicting (102). However, the resolution is also dependent on the leukocytes as neutropenia seems to reduce the capability for thrombus resolution (87).

Future research, both basic laboratory research including studies in murine models, in addition to translational and clinical research, are important to completely bridging the association between inflammation and VTE. This includes a better understanding of the inflammatory aspects of thrombosis and the pathophysiology role of mediator molecules, herein their potential role as diagnostic and prognostic biomarkers. The later will be of special interest for selective patient groups, there biomarkers have demonstrated to be of limited values, including patients with malignant disease and patients with other inflammatory comorbidities. Until then, good clinical judgments combined with established biomarkers and radiological examinations, are still the basic tools in diagnostic approaches for patients with VTE.

## Author contributions

KM: main responsibility for writing; SJ: responsibility for writing of the parts regarding Adhesion Molecules; ØW: responsibility for writing of the parts regarding Matrix Metalloproteases; IN: introduction and illustrations; ØB: main Idea of the publication; HR: corresponding author.

### Conflict of interest statement

The authors declare that the research was conducted in the absence of any commercial or financial relationships that could be construed as a potential conflict of interest.

## References

[1] Cohen AT Agnelli G Anderson FA Arcelus JI Bergqvist D Brecht JG. Venous thromboembolism (VTE) in Europe. The number of VTE events and associated morbidity and mortality. Thromb Haemost. (2007) 98:756–64. 10.1160/TH07-03-0212 17938798

[2] Vaitkus PT Leizorovicz A Cohen AT Turpie AG Olsson CG Goldhaber SZ. Mortality rates and risk factors for asymptomatic deep vein thrombosis in medical patients. Thromb Haemost. (2005) 93:76–9. 10.1160/TH04-05-0323 15630494

[3] Lester W Freemantle N Begaj I Ray D Wood J Pagano D. Fatal venous thromboembolism associated with hospital admission: a cohort study to assess the impact of a national risk assessment target. Heart (2013) 99:1734–9. 10.1136/heartjnl-2013-304479 24038168

[4] Zee RY Glynn RJ Cheng S Steiner L Rose L Ridker PM. An evaluation of candidate genes of inflammation and thrombosis in relation to the risk of venous thromboembolism: The Women's Genome Health Study. Circ Cardiovasc Genet. (2009) 2:57–62. 10.1161/CIRCGENETICS.108.801969 20031567 PMC2747114

[5] Barbar S Noventa F Rossetto V Ferrari A Brandolin B Perlati M. A risk assessment model for the identification of hospitalized medical patients at risk for venous thromboembolism: the Padua Prediction Score. J Thromb Haemost. (2010) 8:2450–7. 10.1111/j.1538-7836.2010.04044.x 20738765

[6] Kahn SR Lim W Dunn AS Cushman M Dentali F Akl EA. Prevention of VTE in nonsurgical patients: antithrombotic therapy and prevention of thrombosis, 9th ed: American college of chest physicians evidence-based clinical practice guidelines. Chest (2012) 141:e195S−226S. 10.1378/chest.11-2296 22315261 PMC3278052

[7] Roumen-Klappe EM Den Heijer M Van Uum SH Van Der Ven-Jongekrijg J Van Der Graaf F Wollersheim H. Inflammatory response in the acute phase of deep vein thrombosis. J Vasc Surg. (2002) 35:701–6. 10.1067/mva.2002.121746 11932666

[8] Duan Q Gong Z Song H Wang L Yang F Lv W. Symptomatic venous thromboembolism is a disease related to infection and immune dysfunction. Int J Med Sci. (2012) 9:453–61. 10.7150/ijms.4453 22859906 PMC3410365

[9] Engelmann B Massberg S. Thrombosis as an intravascular effector of innate immunity. Nat Rev Immunol. (2013) 13:34–45. 10.1038/nri3345 23222502

[10] Lambert MP Sachais BS Kowalska MA. Chemokines and thrombogenicity. Thromb Haemost. (2007) 97:722–9. 10.1160/TH07-01-0046 17479182

[11] Mahindra A Hideshima T Anderson KC. Multiple myeloma: biology of the disease. Blood Rev. (2010) 24(Suppl. 1):S5–11. 10.1016/S0268-960X(10)70003-5 21126636

[12] Turner MD Nedjai B Hurst T Pennington DJ. Cytokines and chemokines: At the crossroads of cell signalling and inflammatory disease. Biochim Biophys Acta (2014) 1843:2563–82. 10.1016/j.bbamcr.2014.05.014 24892271

[13] Wiersinga WJ Leopold SJ Cranendonk DR Van Der Poll T. Host innate immune responses to sepsis. Virulence (2014) 5:36–44. 10.4161/viru.25436 23774844 PMC3916381

[14] Samuels PB Webster DR. The role of venous endothelium in the inception of thrombosis. Ann Surg. (1952) 136:422–38. 14953174 10.1097/00000658-195209000-00010 PMC1802890

[15] Khakpour S Wilhelmsen K Hellman J. Vascular endothelial cell Toll-like receptor pathways in sepsis. Innate Immun. (2015) 21:827–46. 10.1177/1753425915606525 26403174

[16] Nourshargh S Alon R. Leukocyte migration into inflamed tissues. Immunity (2014) 41:694–707. 10.1016/j.immuni.2014.10.008 25517612

[17] Furie B Furie BC. Mechanisms of thrombus formation. N Engl J Med. (2008) 359:938–49. 10.1056/NEJMra0801082 18753650

[18] Von Bruhl ML Stark K Steinhart A Chandraratne S Konrad I Lorenz M. Monocytes, neutrophils, and platelets cooperate to initiate and propagate venous thrombosis in mice *in vivo*. J Exp Med. (2012) 209:819–35. 10.1084/jem.20112322 22451716 PMC3328366

[19] Brill A Fuchs TA Chauhan AK Yang JJ De Meyer SF Kollnberger M. von Willebrand factor-mediated platelet adhesion is critical for deep vein thrombosis in mouse models. Blood (2011) 117:1400–7. 10.1182/blood-2010-05-287623 20959603 PMC3056477

[20] Payne H Ponomaryov T Watson SP Brill A. Mice with a deficiency in CLEC-2 are protected against deep vein thrombosis. Blood (2017) 129:2013–20. 10.1182/blood-2016-09-742999 28104688 PMC5408561

[21] Wang Y Gao H Shi C Erhardt PW Pavlovsky A A Soloviev D. Leukocyte integrin Mac-1 regulates thrombosis via interaction with platelet GPIbalpha. Nat Commun. (2017) 8:15559. 10.1038/ncomms15559 28555620 PMC5477519

[22] Manfredi AA Ramirez GA Rovere-Querini P Maugeri N. The Neutrophil's choice: phagocytose vs make neutrophil extracellular traps. Front Immunol. (2018) 9:288. 10.3389/fimmu.2018.00288 29515586 PMC5826238

[23] Jimenez-Alcazar M Rangaswamy C Panda R Bitterling J Simsek YJ Long AT. Host DNases prevent vascular occlusion by neutrophil extracellular traps. Science (2017) 358:1202–6. 10.1126/science.aam8897 29191910

[24] Nissinen L Kahari VM. Matrix metalloproteinases in inflammation. Biochim Biophys Acta (2014) 1840:2571–80. 10.1016/j.bbagen.2014.03.007 24631662

[25] Dinarello CA Van Der Meer JW. Treating inflammation by blocking interleukin-1 in humans. Semin Immunol. (2013) 25:469–84. 10.1016/j.smim.2013.10.008 24275598 PMC3953875

[26] Narayanan KB Park HH. Toll/interleukin-1 receptor (TIR) domain-mediated cellular signaling pathways. Apoptosis (2015) 20:196–209. 10.1007/s10495-014-1073-1 25563856

[27] Schaper F Rose-John S. Interleukin-6: Biology, signaling and strategies of blockade. Cytokine Growth Factor Rev. (2015) 26:475–87. 10.1016/j.cytogfr.2015.07.004 26189695

[28] Vignali DA Kuchroo VK. IL-12 family cytokines: immunological playmakers. Nat Immunol. (2012) 13:722–8. 10.1038/ni.2366 22814351 PMC4158817

[29] Dennis KL Blatner NR Gounari F Khazaie K. Current status of interleukin-10 and regulatory T-cells in cancer. Curr Opin Oncol. (2013) 25:637–45. 10.1097/CCO.0000000000000006 24076584 PMC4322764

[30] Mackenzie KF Pattison MJ Arthur JS. Transcriptional regulation of IL-10 and its cell-specific role *in vivo*. Crit Rev Immunol. (2014) 34:315–45. 10.1615/CritRevImmunol.2014010694 24941159

[31] Walter MR. The molecular basis of IL-10 function: from receptor structure to the onset of signaling. Curr Top Microbiol Immunol. (2014) 380:191–212. 10.1007/978-3-662-43492-5_9 25004819 PMC4489423

[32] Ni G Wang T Walton S Zhu B Chen S Wu X. Manipulating IL-10 signalling blockade for better immunotherapy. Cell Immunol. (2015) 293:126–9. 10.1016/j.cellimm.2014.12.012 25596475

[33] Zonneveld R Martinelli R Shapiro NI Kuijpers TW Plotz FB Carman CV. Soluble adhesion molecules as markers for sepsis and the potential pathophysiological discrepancy in neonates, children and adults. Crit Care (2014) 18:204. 10.1186/cc13733 24602331 PMC4014977

[34] Hatfield KJ Reikvam H Bruserud O. The crosstalk between the matrix metalloprotease system and the chemokine network in acute myeloid leukemia. Curr Med Chem. (2010) 17:4448–61. 10.2174/092986710794183033 21062258

[35] Vandenbroucke RE Libert C. Is there new hope for therapeutic matrix metalloproteinase inhibition? Nat Rev Drug Discov. (2014) 13:904–27. 10.1038/nrd4390 25376097

[36] Beckers MM Ruven HJ Haas FJ Doevendans PA Ten Cate H Prins MH. Single nucleotide polymorphisms in inflammation-related genes are associated with venous thromboembolism. Eur J Intern Med. (2010) 21:289–92. 10.1016/j.ejim.2010.04.001 20603037

[37] Christiansen SC Naess IA Cannegieter SC Hammerstrom J Rosendaal FR Reitsma PH. Inflammatory cytokines as risk factors for a first venous thrombosis: a prospective population-based study. PLoS Med. (2006) 3:e334. 10.1371/journal.pmed.0030334 16933968 PMC1551920

[38] Van Minkelen R De Visser MC Houwing-Duistermaat JJ Vos HL Bertina RM Rosendaal FR. Haplotypes of IL1B, IL1RN, IL1R1, and IL1R2 and the risk of venous thrombosis. Arterioscler Thromb Vasc Biol. (2007) 27:1486–91. 10.1161/ATVBAHA.107.140384 17413037

[39] Malaponte G Polesel J Candido S Sambataro D Bevelacqua V Anzaldi M. IL-6-174 G > C and MMP-9-1562 C > T polymorphisms are associated with increased risk of deep vein thrombosis in cancer patients. Cytokine (2013) 62:64–9. 10.1016/j.cyto.2013.02.017 23490413

[40] Matos MF Lourenco DM Orikaza CM Bajerl JA Noguti MA Morelli VM. The role of IL-6, IL-8 and MCP-1 and their promoter polymorphisms IL-6−174GC, IL-8−251AT and MCP-1−2518AG in the risk of venous thromboembolism: a case-control study. Thromb Res. (2011) 128:216–20. 10.1016/j.thromres.2011.04.016 21620438

[41] Vormittag R Hsieh K Kaider A Minar E Bialonczyk C Hirschl M. Interleukin-6 and interleukin-6 promoter polymorphism (-174) G > C in patients with spontaneous venous thromboembolism. Thromb Haemost. (2006) 95:802–6. 10.1160/TH05-12-0816 16676071

[42] Mahemuti A Abudureheman K Aihemaiti X Hu XM Xia YN Tang BP. Association of interleukin-6 and C-reactive protein genetic polymorphisms levels with venous thromboembolism. Chin Med J. (2012) 125:3997–4002. 10.3760/cma.j.issn.0366-6999.2012.22.016 23158132

[43] Yadav U Mahemuti A Hu X Abudureheman K Xia Y Tang B. Single nucleotide polymorphisms in interleukin-6 and their association with venous thromboembolism. Mol Med Rep. (2015) 11:4664–70. 10.3892/mmr.2015.3248 25625484

[44] Matsuo K Hasegawa K Yoshino K Murakami R Hisamatsu T Stone RL. Venous thromboembolism, interleukin-6 and survival outcomes in patients with advanced ovarian clear cell carcinoma. Eur J Cancer (2015) 51:1978–88. 10.1016/j.ejca.2015.07.012 26238017 PMC7304744

[45] Lim SH Woo SY Kim S Ko YH Kim WS Kim SJ. Cross-sectional study of patients with diffuse large b-cell lymphoma: assessing the effect of host status, Tumor Burden, and inflammatory activity on venous thromboembolism. Cancer Res Treat. (2016) 48:312–21. 10.4143/crt.2014.266 25761485 PMC4720058

[46] Matos MF Lourenco DM Orikaza CM Gouveia CP Morelli VM. Abdominal obesity and the risk of venous thromboembolism among women: a potential role of interleukin-6. Metab Syndr Relat Disord. (2013) 11:29–34. 10.1089/met.2012.0077 23025692

[47] Jezovnik MK Poredos P. Idiopathic venous thrombosis is related to systemic inflammatory response and to increased levels of circulating markers of endothelial dysfunction. Int Angiol. (2010) 29:226–31. 20502419

[48] De Franciscis S Gallelli L Amato B Butrico L Rossi A Buffone G. Plasma MMP and TIMP evaluation in patients with deep venous thrombosis: could they have a predictive role in the development of post-thrombotic syndrome? Int Wound J. (2016) 13:1237–45. 10.1111/iwj.12489 26403997 PMC7949807

[49] Du T Tan Z. Relationship between deep venous thrombosis and inflammatory cytokines in postoperative patients with malignant abdominal tumors. Braz J Med Biol Res. (2014) 47:1003–7. 10.1590/1414-431X20143695 25296364 PMC4230292

[50] Wik HS Jacobsen AF Mowinckel MC Sandset PM. The role of inflammation in post-thrombotic syndrome after pregnancy-related deep vein thrombosis: a population-based, cross-sectional study. Thromb Res. (2016) 138:16–21. 10.1016/j.thromres.2015.12.014 26826503

[51] Bittar LF Mazetto Bde M Orsi FL Collela MP De Paula EV Annichino-Bizzacchi JM. Long-term increased factor VIII levels are associated to interleukin-6 levels but not to post-thrombotic syndrome in patients with deep venous thrombosis. Thromb Res. (2015) 135:497–501. 10.1016/j.thromres.2014.12.024 25575413

[52] Van Aken BE Den Heijer M Bos GM Van Deventer SJ Reitsma PH. Recurrent venous thrombosis and markers of inflammation. Thromb Haemost. (2000) 83:536–9. 10.1055/s-0037-1613858 10780312

[53] Jezovnik MK Poredos P. Factors influencing the recanalisation rate of deep venous thrombosis. Int Angiol. (2012) 31:169–75. 22466983

[54] Wojcik BM Wrobleski SK Hawley AE Wakefield TW Myers D. D. Jr. Diaz JA. Interleukin-6: a potential target for post-thrombotic syndrome. Ann Vasc Surg. (2011) 25:229–39. 10.1016/j.avsg.2010.09.003 21131172

[55] Shbaklo H Holcroft CA Kahn SR. Levels of inflammatory markers and the development of the post-thrombotic syndrome. Thromb Haemost. (2009) 101:505–12. 10.1160/TH08-08-0511 19277412

[56] Roumen-Klappe EM Janssen MC Van Rossum J Holewijn S Van Bokhoven MM Kaasjager K. Inflammation in deep vein thrombosis and the development of post-thrombotic syndrome: a prospective study. J Thromb Haemost. (2009) 7:582–7. 10.1111/j.1538-7836.2009.03286.x 19175493

[57] Jezovnik MK Fareed J Poredos P. Patients With a History of Idiopathic Deep Venous thrombosis have long-term increased levels of inflammatory markers and markers of endothelial damage. Clin Appl Thromb Hemost. (2017) 23:124–31. 10.1177/1076029616670259 27663463

[58] Rabinovich A Cohen JM Cushman M Wells PS Rodger MA Kovacs MJ. Inflammation markers and their trajectories after deep vein thrombosis in relation to risk of post-thrombotic syndrome. J Thromb Haemost. (2015) 13:398–408. 10.1111/jth.12814 25495610

[59] Van Aken BE Reitsma PH Rosendaal FR. Interleukin 8 and venous thrombosis: evidence for a role of inflammation in thrombosis. Br J Haematol. (2002) 116:173–7. 10.1046/j.1365-2141.2002.03245.x 11841414

[60] Montes-Worboys A Arellano E Elias T Leon J Rodriguez-Portal JA Otero R. Residual thrombosis after a first episode of proximal deep venous thrombosis. Blood Coagul Fibrinolysis (2013) 24:335–60. 10.1097/MBC.0b013e32835c32ef 23314382

[61] Proctor MC Sullivan V Zajkowski P Wolk SW Pomerantz RA Wakefield TW. A role for interleukin-10 in the assessment of venous thromboembolism risk in injured patients. J Trauma (2006) 60:147–51. 10.1097/01.ta.0000197180.79965.bc 16456448

[62] Tang B Chen YK Luo WJ Fu J Sun JM. Association between interleukin-10−1082A/G,−819C/T and−592C/A polymorphisms with deep venous thrombosis. Hum Immunol. (2014) 75:203–7. 10.1016/j.humimm.2013.12.013 24374045

[63] Ferroni P Riondino S Portarena I Formica V La Farina F Martini F. Association between increased tumor necrosis factor alpha levels and acquired activated protein C resistance in patients with metastatic colorectal cancer. Int J Colorectal Dis. (2012) 27:1561–7. 10.1007/s00384-012-1493-8 22581210

[64] Roselli M Ferroni P Rolfo C Peeters M Palmirotta R Formica V. TNF-alpha gene promoter polymorphisms and risk of venous thromboembolism in gastrointestinal cancer patients undergoing chemotherapy. Ann Oncol. (2013) 24:2571–5. 10.1093/annonc/mdt251 23852308

[65] Horakova K Chylkova A Kolorz M Bartosova L Pechacek V Starostka D. Polymorphism G-308A in the promoter of the tumor necrosis factor-alpha gene and its association with the risk of venous thromboembolism. Blood Coagul Fibrinolysis (2012) 23:316–9. 10.1097/MBC.0b013e3283527506 22473048

[66] Nosaka M Ishida Y Kimura A Kuninaka Y Inui M Mukaida N. Absence of IFN-gamma accelerates thrombus resolution through enhanced MMP-9 and VEGF expression in mice. J Clin Invest. (2011) 121:2911–20. 10.1172/JCI40782 21646723 PMC3223815

[67] Malarstig A Eriksson P Rose L Diehl KA Hamsten A Ridker PM. Genetic variants of tumor necrosis factor superfamily, member 4 (TNFSF4), and risk of incident atherothrombosis and venous thromboembolism. Clin Chem. (2008) 54:833–40. 10.1373/clinchem.2007.096479 18356244

[68] Memon AA Sundquist K Wang X Svensson PJ Sundquist J Zoller B. Transforming growth factor (TGF)-beta levels and unprovoked recurrent venous thromboembolism. J Thromb Thrombolysis (2014) 38:348–54. 10.1007/s11239-013-1047-0 24402195

[69] Mosevoll KA Lindas R Tvedt TH Bruserud O Reikvam H. Altered plasma levels of cytokines, soluble adhesion molecules and matrix metalloproteases in venous thrombosis. Thromb Res. (2015) 136:30–9. 10.1016/j.thromres.2015.04.002 25895848

[70] Heit JA Spencer FA White RH. The epidemiology of venous thromboembolism. J Thromb Thrombolysis (2016) 41:3–14. 10.1007/s11239-015-1311-6 26780736 PMC4715842

[71] Morange PE Oudot-Mellakh T Cohen W Germain M Saut N Antoni G. KNG1 Ile581Thr and susceptibility to venous thrombosis. Blood (2011) 117:3692–4. 10.1182/blood-2010-11-319053 21270443

[72] Croles FN Nasserinejad K Duvekot JJ Kruip MJ Meijer K Leebeek FW. Pregnancy, thrombophilia, and the risk of a first venous thrombosis: systematic review and bayesian meta-analysis. BMJ (2017) 359:j4452. 10.1136/bmj.j4452 29074563 PMC5657463

[73] Bezemer ID Van Der Meer FJ Eikenboom JC Rosendaal FR Doggen CJ. The value of family history as a risk indicator for venous thrombosis. Arch Intern Med. (2009) 169:610–5. 10.1001/archinternmed.2008.589 19307525

[74] Wakefield TW Greenfield LJ Rolfe MW Delucia A. III Strieter RM Abrams GD. Inflammatory and procoagulant mediator interactions in an experimental baboon model of venous thrombosis. Thromb Haemost. (1993) 69:164–72. 8456429

[75] Antonopoulos CN Sfyroeras GS Kakisis JD Moulakakis KG Liapis CD. The role of soluble P selectin in the diagnosis of venous thromboembolism. Thromb Res. (2013) 133:17–24. 10.1016/j.thromres.2013.08.014 24012101

[76] Vandy FC Stabler C Eliassen AM Hawley AE Guire KE Myers DD. Soluble P-selectin for the diagnosis of lower extremity deep venous thrombosis. J Vasc Surg Venous Lymphat Disord. (2013) 1:117–25. 10.1016/j.jvsv.2012.09.001 23998134 PMC3752921

[77] Ramacciotti E Myers DD Jr Wrobleski SK Deatrick KB Londy FJ Rectenwald JE. P-selectin/ PSGL-1 inhibitors versus enoxaparin in the resolution of venous thrombosis: a meta-analysis. Thromb Res. (2010) 125:e138–42. 10.1016/j.thromres.2009.10.022 19962723 PMC2942795

[78] Ramacciotti E Blackburn S Hawley AE Vandy F Ballard-Lipka N Stabler C. Evaluation of soluble P-selectin as a marker for the diagnosis of deep venous thrombosis. Clin Appl Thromb Hemost. (2011) 17:425–31. 10.1177/1076029611405032 21593019 PMC3306250

[79] Uitte De Willige S De Visser MC Vos HL Houwing-Duistermaat JJ Rosendaal FR Bertina RM. Selectin haplotypes and the risk of venous thrombosis: influence of linkage disequilibrium with the factor V Leiden mutation. J Thromb Haemost. (2008) 6:478–85. 10.1111/j.1538-7836.2007.02879.x 18182036

[80] Deatrick KB Elfline M Baker N Luke CE Blackburn S Stabler C. Postthrombotic vein wall remodeling: preliminary observations. J Vasc Surg. (2011) 53:139–46. 10.1016/j.jvs.2010.07.043 20869834 PMC3010467

[81] Rectenwald JE Myers DD Jr Hawley AE Longo C Henke PK Guire KE. D-dimer, P-selectin, and microparticles: novel markers to predict deep venous thrombosis. A pilot study. Thromb Haemost (2005) 94:1312–7. 10.1160/TH05-06-0426 16411411

[82] Bucek RA Reiter M Quehenberger P Minar E Baghestanian M. The role of soluble cell adhesion molecules in patients with suspected deep vein thrombosis. Blood Coagul Fibrinolysis (2003) 14:653–7. 10.1097/00001721-200310000-00006 14517490

[83] Bozic M Blinc A Stegnar M. D-dimer, other markers of haemostasis activation and soluble adhesion molecules in patients with different clinical probabilities of deep vein thrombosis. Thromb Res. (2002) 108:107–14. 10.1016/S0049-3848(03)00007-0 12590945

[84] Yang LC Wang CJ Lee TH Lin FC Yang BY Lin CR. Early diagnosis of deep vein thrombosis in female patients who undergo total knee arthroplasty with measurement of P-selectin activation. J Vasc Surg. (2002) 35:707–12. 10.1067/mva.2002.121852 11932667

[85] Blann AD Noteboom WM Rosendaal FR. Increased soluble P-selectin levels following deep venous thrombosis: cause or effect? Br J Haematol. (2000) 108:191–3. 10.1046/j.1365-2141.2000.01813.x 10651744

[86] Gremmel T Ay C Seidinger D Pabinger I Panzer S Koppensteiner R. Soluble p-selectin, D-dimer, and high-sensitivity C-reactive protein after acute deep vein thrombosis of the lower limb. J Vasc Surg. (2011) 54:48S−55S. 10.1016/j.jvs.2011.05.097 21890302

[87] Thanaporn P Myers DD Wrobleski SK Hawley AE Farris DM Wakefield TW. P-selectin inhibition decreases post-thrombotic vein wall fibrosis in a rat model. Surgery (2003) 134:365–71. 10.1067/msy.2003.249 12947342

[88] Myers DD Jr Henke PK Wrobleski SK Hawley AE Farris DM Chapman AM. P-selectin inhibition enhances thrombus resolution and decreases vein wall fibrosis in a rat model. J Vasc Surg. (2002) 36:928–38. 10.1067/mva.2002.128636 12422103

[89] Mosevoll KA Lindas R Wendelbo O Bruserud O Reikvam H. Systemic levels of the endothelium-derived soluble adhesion molecules endocan and E-selectin in patients with suspected deep vein thrombosis. Springerplus (2014) 3:571. 10.1186/2193-1801-3-571 25332871 PMC4197195

[90] Henke PK. Plasmin and matrix metalloproteinase system in deep venous thrombosis resolution. Vascular (2007) 15:366–71. 10.2310/6670.2007.00050 18053422

[91] Goodwin S Mcpherson JD Mccombie WR. Coming of age: ten years of next-generation sequencing technologies. Nat Rev Genet. (2016) 17:333–51. 10.1038/nrg.2016.49 27184599 PMC10373632

[92] Connors JM. Thrombophilia testing and venous thrombosis. N Engl J Med. (2017) 377:1177–87. 10.1056/NEJMra1700365 28930509

[93] Carr JA Cho JS. Low molecular weight heparin suppresses tumor necrosis factor expression from deep vein thrombosis. Ann Vasc Surg. (2007) 21:50–5. 10.1016/j.avsg.2006.07.003 17349336

[94] Moaveni DK Lynch EM Luke C Sood V Upchurch GR Wakefield TW. Vein wall re-endothelialization after deep vein thrombosis is improved with low-molecular-weight heparin. J Vasc Surg. (2008) 47:616–24. 10.1016/j.jvs.2007.11.040 18295113 PMC2350236

[95] Rodriguez AL Wojcik BM Wrobleski SK Myers D. D. Jr. Wakefield TW Diaz JA. Statins, inflammation and deep vein thrombosis: a systematic review. J Thromb Thrombolysis (2012) 33:371–82. 10.1007/s11239-012-0687-9 22278047 PMC3338886

[96] Zolcinski M Ciesla-Dul M Potaczek DP Undas A. Atorvastatin favorably modulates proinflammatory cytokine profile in patients following deep vein thrombosis. Thromb Res. (2013) 132:e31–5. 10.1016/j.thromres.2013.04.026 23791132

[97] Feng Y Lei B Zhang F Niu L Zhang H Zhang M. Anti-inflammatory effects of simvastatin during the resolution phase of experimentally formed venous thrombi. J Investig Med. (2017) 65:999–1007. 10.1136/jim-2017-000442 28442532

[98] Henke PK Wakefield TW Kadell AM Linn MJ Varma MR Sarkar M. Interleukin-8 administration enhances venous thrombosis resolution in a rat model. J Surg Res. (2001) 99:84–91. 10.1006/jsre.2001.6122 11421608

[99] Wang ZW Wang JJ Zhang JZ Xue ZJ Miao J Li L. Thrombolysis of deep vein thrombosis and inhibiting chemotaxis of macrophage by MCP-1 blockage. Eur Rev Med Pharmacol Sci. (2017) 21:1695–701. 28429334

[100] Varma MR Varga AJ Knipp BS Sukheepod P Upchurch GR Kunkel SL. Neutropenia impairs venous thrombosis resolution in the rat. J Vasc Surg. (2003) 38:1090–8. 10.1016/S0741-5214(03)00431-2 14603221

[101] Henke PK Pearce CG Moaveni DM Moore AJ Lynch EM Longo C. Targeted deletion of CCR2 impairs deep vein thombosis resolution in a mouse model. J Immunol. (2006) 177:3388–97. 10.4049/jimmunol.177.5.3388 16920980

[102] Varma MR Moaveni DM Dewyer NA Varga AJ Deatrick KB Kunkel SL. Deep vein thrombosis resolution is not accelerated with increased neovascularization. J Vasc Surg. (2004) 40:536–42. 10.1016/j.jvs.2004.05.023 15337885

